# Molecular characterization of circulating tumour cells identifies predictive markers for outcome in primary, triple‐negative breast cancer patients

**DOI:** 10.1111/jcmm.15349

**Published:** 2020-06-18

**Authors:** Ann‐Kathrin Bittner, Corinna Keup, Oliver Hoffmann, Siegfried Hauch, Rainer Kimmig, Sabine Kasimir‐Bauer

**Affiliations:** ^1^ Department of Gynecology and Obstetrics University Hospital of Essen Essen Germany; ^2^ QIAGEN GmbH Hilden Germany

**Keywords:** breast cancer, circulating tumour cells, RNA profiles, triple‐negative breast cancer

## Abstract

mRNA profiles of circulating tumour cells (CTCs) were analysed in patients with triple‐negative breast cancer (TNBC) (pts) before (BT) and after therapy (AT) to identify additional treatment options. 2 × 5 mL blood of 51 TNBC pts and 24 non‐TNBC pts (HR+/HER2−; HR−/HER2+) was analysed for CTCs using the AdnaTest EMT‐2/Stem Cell Select™, followed by mRNA isolation and cDNA analysis for 17 genes by qPCR *PIK3CA*, *AKT2*, *MTOR* and the resistance marker *AURKA* and *ERCC1* were predominantly expressed in all breast cancer subtypes, the latter ones especially AT. In TNBC pts, *ERBB3, EGFR, SRC, NOTCH, ALK* and *AR* were uniquely present and *ERBB2+/ERBB3* + CTCs were found BT and AT in about 20% of cases. *EGFR+/ERBB2+/ERBB3 + *CTCs BT and *ERBB2+/ERBB3 + *CTCs AT significantly correlated with a shorter progression‐free survival (PFS; *P* = 0.01 and *P* = 0.02). Platinum‐based therapy resulted in a reduced PFS (*P* = 0.02) and an induction of *PIK3CA* expression in CTCs AT. In non‐TNBC pts, BT, the expression pattern in CTCs was similar. AURKA+/ERCC1 + CTCs were found in 40% of HR−/HER2 + pts BT and AT. In the latter group, *NOTCH, PARP1* and *SRC1* were only present AT and *ERBB2 + *CTCs completely disappeared AT. These findings might help to predict personalized therapy for TNBC pts in the future.

## 1 INTRODUCTION

Breast cancer (BC) is the most common cancer in women worldwide and even though the 5‐year overall survival (OS) rate reaches 88% in Germany,^1^ in about 20% of patients,^2^ in the so‐called triple‐negative breast cancer (TNBC) patients with the absence of the hormonal receptors as well as the growth factor receptor HER2 on the primary tumour, long‐term outcome is poor.^3^ Up to now, in most cases, neoadjuvant chemotherapy (NACT) is the standard of care for TNBC,^4^, ^5^ and since 2016, the AGO (Arbeitsgemeinschaft Gynäkologische Onkologie e.V.) has recommended a combination therapy containing carboplatin ^6^ which improved the pathological complete response (pCR) rate ^7^ as well as the event‐free survival and overall survival (OS) in some neoadjuvant trials.^8^, ^9^ Response to therapy in the neoadjuvant setting is assessed as pCR, which is achieved in about 27%‐45% of the patients depending on tumour stage and which was shown to improve prognosis in some clinical studies.^2^, ^3^, ^10^, ^11^, ^12^, ^13^, ^14^ Those patients that do not achieve a pCR after NACT have a worse OS which might be improved by the addition of capecitabine after NACT, resulting in prolonged progression‐free survival (PFS) and OS rates as shown in the CreateX trial.^15^ However, despite achieving a pCR, about 5%‐20% of patients will experience relapse.^14^

Up to now, except for the pCR rate, the success or failure of anti‐cancer therapies in BC is only assessed retrospectively by the absence or presence of overt metastases during the post‐operative follow‐up period. Therefore, other markers are needed to identify patients at high risk of recurrence to offer additional therapeutic options.

Circulating tumour cells (CTCs), the precursor of metastatic disease, would be an ideal surrogate marker to identify prognostic and predictive factors directly at primary diagnosis to guide optimal individual therapeutic strategies for metastasis prevention. CTCs in primary BC have been extensively studied in large patient cohorts and hold strong potential to be translated into individual targeted therapy since their prognostic significance with regard to reduced PFS and OS has already been demonstrated.^16^, ^17^, ^18^ Although their frequency in the primary setting is quite low, a variety of methods have been established to detect and characterize CTCs.^19^, ^20^ CTCs were shown to be very heterogeneous, even within the same patient, including the presence and persistence of resistant and stem cell like CTCs as well as CTCs in epithelial mesenchymal transition (EMT).^21^, ^22^, ^23^, ^24^ Furthermore, a discordant receptor status between ER, PR and especially HER2 on CTCs and the primary tumour has been demonstrated which explains why CTCs survive after targeted therapy based on markers detected on the primary tumour.^25^, ^26^, ^27^

It might be assumed that CTCs may, therefore, also represent distinct metabolic profiles for survival, metastatic spread and therapy resistance since the prognosis in BC has been shown to be different in various BC subtypes.^3^ For patients with TNBC, CTC counts were shown to have prognostic value in early as well as later stages of the disease,^28^, ^29^, ^30^ but characteristics of these cells have rarely been shown. In this regard, our group demonstrated that CTCs found in primary, non‐TNBC patients were frequently characterized as triple‐negative phenotype regardless of the ER, PR and HER2 status of the primary tumour.^25^ On the other hand, it was recently demonstrated that CTCs of early‐stage TNBC patients frequently expressed ER, PR, HER2 and EGFR with a predomination of the latter one over the other phenotypes. Triple‐staining experiments revealed that distinct subpopulations were identified in individual patients.^31^ These data emphasize that a comprehensive characterization of these cells is essential to find targets for additional treatment options in this high‐risk BC subgroup.

In the present study, therefore, we aimed to analyse mRNA profiles of CTCs from primary, non‐metastatic TNBC patients before and after NACT using a multimarker gene panel to (a) identify additional individualized treatment options, (b) identify predictive markers for treatment outcome and (c) compare the results with CTCs of non‐TNBC patients.

## 2 PATIENTS AND METHODS

### 2.1 Patient population and patient characteristics

The study was conducted in the Department of Gynecology and Obstetrics, at the University Hospital of Essen. In total, 75 BC patients (51 TNBC patients [BT: n = 39, AT: n = 37]; 24 non‐TNBC patients [BT: n = 20, AT: n = 21], including HR+/HER2− patients [n = 14; 12 BT and 13 AT, respectively] and HR−/HER2+ patients [n = 10; 8 BT and 8 AT, respectively]), diagnosed between January 2013 and March 2016, were enrolled. All specimens were obtained and collected after written informed consent from all subjects using protocols approved by the clinical ethic committee of the University Hospital Essen (05/2856). Patient characteristics are documented in Table 1.

**Table 1:** Clinical data of patients

	Total (%)	TNBC Total (%/% of all applicable/known)	HR+/HER2 – Total (%/% of all applicable/known)	HR−/HER2 + Total (%/% of all applicable/known)
Total	75 (100)	51 (68.00)	14 (18.67)	10 (13.33)
Age (years)	50	51	51	47
<60	57 (76/76)	37 (72/72)	12 (86(86)	8 (80/80)
>60	18 (24/24)	14 (27/27)	2 (14/14)	2 (20/20)
Menopausal Status				
Premenopausal	23 (31/31)	13 (25/25)	6 (43/43)	4 (40/44)
Perimenopausal	8 (11/11)	7 (14/14)	1 (7/7)	0 (0)
Postmenopausal	43 (57/58)	31 (61/61)	7 (50/50)	5 (50/56)
Nk	1 (1/x)	0 (0/x)	0 (0/x)	1 (10/x)
Histology				
Ductal	54 (72/76)	36 (71/75)	10 (71/71)	8 (80/89)
Lobular	5 (7/7)	0 (0/0)	4 (29/29)	1 (10/11)
Others	12 (16/17)	12 (24/25)	0 (0/0)	0 (0/0)
nk	4 (5/x)	3 (8/x)	0 (0/x)	1 (10/x)
Grading				
I	0 (0/0)	0 (0/0)	0 (0/0)	0 (0)
II	20 (27/27)	8 (16/16)	10 (71/71)	2 (20/20)
III	54 (72/73)	42 (82/84)	4 (29/29)	8 (80/80)
nk	1 (1/x)	1 (2/x)	0 (0/x)	0 (0/x)
Ki 67 (%)				
<15	3 (4/5)	3 (6/7)	0 (0/0)	0 (0/0)
15‐30	14 (19/22)	4 (8/10)	7 (50/50)	3 (30/33)
>30	47 (63/73)	34 (67/83)	7 (50/50)	6 (60/67)
nk	11 (15/x)	10 (37/x)	0 (0/0)	1 (10/x)
Tumour size at first diagnosis (c/pT)				
T1	32 (43/43)	20 (39/39)	9 (64/64)	3 (30/30)
T2	35 (47/43)	27 (53/53)	3 (21/21)	5 (50/50)
T3‐4	8 (11/11)	4 (8/8)	2 (14/14)	2 (20/20)
nk	0 (0/x)	0 (0/x)	0 (0/x)	0 (0/x)
Tumour size after NACT				
ypT0	31 (41/45)	20 (39/43)	4 (29/31)	7 (70/78)
ypT1	24 (32/35)	15 (29/32)	7 (50/54)	2 (20/22)
ypT2	12 (16/17)	10 (20/21)	2 (14/15)	0 (0/0)
ypT3‐4	2 (3/3)	2 (4/4)	0 (0/0)	0 (0/0)
nk	1 (1/x)	0 (0/x)	1 (7/x)	0 (0/x)
na	5 (7/x)	4 (84/x)	0 (0/x)	1 (10/x)
Nodal status (c/pN)				
Node negative	47 (63/63)	37 (73/73)	8 (57/57)	2 (20/20)
Node positive	28 (37/37)	14 (27/27)	6 (43/43)	8 (80/80)
N1	18 (24/24)	9 (18/18)	3 (21/21)	6 (60/60)
N2	4 (5/5)	1 (2/2)	2 (14/14)	1 (10/10)
N3	6 (8/8)	4 (8/8)	1 (7/7)	1 (10/10)
nk	0 (0/x)	0 (0/x)	0 (0/x)	0 (0/x)
Nodal status (pN)				
Node negative	4(5/67)	2 (4/50)	0 (0/0)	2 (20/100)
Node positive	2 (3/33)	2 (4/50)	0 (0/0)	0 (0/0)
ypN1	1 (1/17)	1 (2/25)	0 (0/0)	0 (0/0)
ypN2	1 (1/17)	1 (2/25)	0 (0/0)	0 (0/0)
ypN3	0 (0/0)	0 (0/0)	0 (0/0)	0 (0/0)
Nk	0 (0/0)	0 (0/0)	0 (0/0)	0 (0/0)
na	69 (92/x)	47 (95/x)	14 (100/x)	8 (80/x)
Pathological response				
Response	64 (85/93)	44 (86/94)	12 (86/93)	8 (80/89)
Complete response	32 (43/46)	21 (41/45)	4 (29/31)	7 (70/78)
Partial response	32 (43/46)	23 (45/49)	8 (57/62)	1 (10/11)
No response	5 (7/7)	3 (6/6)	1 (7/8)	1 (10/11)
Nk	1 (1/x)	0 (0/0)	1 (7/x)	0 (0/0)
Na	5 (7/x).	4 (8/x)	0 (0/x)	1 (10/x)
Chemotherapy				
Yes	74 (99)	51 (100)	14 (59)	9 (90/90)
Neoadjuvant	69 (92/93)	47 (92/92)	14 (44)	8 (80/89)
Adjuvant	5 (7/7)	4 (8/8)	0 (15)	1(0/0)
nd	1 (1/x)	0 (0/x)	0 (0/x)	1 (10/11)

Note

c (clinical)/p (pathological) T (tumour size), c/pN (nodal status), BT (before therapy), AT (after therapy).

Abbreviations: na, not applicable; nd, not done; nk, not known.

### 2.2 Eligibility criteria and response criteria

The eligibility criteria were as follows: histologically proven BC, blood samples obtained at the time of primary diagnosis and after NACT, if applicable, no severe uncontrolled comorbidities or medical conditions, and no further malignancies at present or in the patient history, completion of neoadjuvant or adjuvant treatment according to current guidelines^4^ including NACT (anthracyclines, taxanes, cyclophosphamide, carboplatin, myocet, gemcitabine), anti‐hormonal therapy in the case of hormone responsive tumours (tamoxifen or an aromatase inhibitor), Herceptin and Perjeta in the case of HER2 positivity and radiotherapy. For each of the 75 patients, the tumour type, TNM‐staging, grading and KI67 were assessed in the Institute of Pathology, at the University Hospital Essen as part of the West German Comprehensive Cancer Center. Pathological response to therapy was defined according to the grading system of Sinn and colleagues: (Sinn = 0, no pathological response; Sinn = 1‐3, pathological partial response [pPR]; Sinn = 1: resorption and tumour sclerosis, Sinn = 2: minimal residual invasive tumour [<0.5 cm]; Sinn = 3: residual non‐invasive tumour only, ductal carcinoma in situ [DCIS]; and Sinn = 4, pathological complete response [pCR], no evidence of residual invasive cancer and DCIS, both, in breast and axilla).^32^

### 2.3 Sampling of blood

2 × 5 mL EDTA blood was collected for CTC isolation in S‐Monovettes^®^ (Sarstedt AG & Co., Nümbrecht, Germany) BT (n = 59 patients) and AT, before surgery (n = 58 patients). The samples were stored at 4°C and were processed not later than 4 hours after blood withdrawal.

### 2.4 Enrichment of circulating tumour cells

CTCs were isolated from 2 × 5 mL blood by positive immunomagnetic selection targeting EpCAM, EGFR and HER2 (AdnaTest EMT‐2/StemCell Select™, QIAGEN GmbH, Hilden, Germany). The method has been described in detail elsewhere.^33^ Briefly, labelled CTCs were extracted using a magnetic particle concentrator and were lysed according to the manufacturer's instructions. Cell lysates were stored for a maximum of 2 weeks at –80°C until further processing.

### 2.5 mRNA isolation and reverse transcription

mRNA was isolated from the cell lysates by oligo(dT)_25_‐coated magnetic beads. Purified mRNA was reverse‐transcribed (AdnaTest EMT‐2/StemCell Detect™, QIAGEN GmbH, Hilden, Germany) with a final reaction volume of 40 µL, and cDNA was stored at −20°C.^33^

### 2.6 Quantitative PCR

The AdnaTest TNBC Panel prototype with a detection limit down to 2 cells/5 mL blood consists of in‐house‐designed multimarker RT‐qPCR assays (QIAGEN GmbH, Germany) and required transcript‐specific preamplification of 6.25 µL cDNA using the TATAA Multiplex Grand Master Mix (TATAA Biocenter AB, Sweden) with 18 PCR cycles. Preamplified cDNA (2 µL; 1:10 diluted) was analysed in duplicates for one of the 18 transcripts (namely *AKT2, ALK, AR, AURKA, BRCA1, EGFR, ERCC1, ERBB2, ERBB3, KIT, KRT5, MET, MTOR, NOTCH1, PARP1, PIK3CA, SRC and GAPDH*) in a reaction volume with SYBR Green‐based components of in total 10 µL. Additionally to fluorescence readout in each cycle, melting curves were obtained. The method has been recently published in detail.^33^

### 2.7 Data evaluation

CTC isolation was conducted in duplicate from 2 × 5 mL blood for each patient sample. cDNA was analysed separately from these duplicates. After binary evaluation of the qPCR data, described below, signals per patient were regarded positive if at least one of the sample duplicates showed a positive ∆(∆)C_q_ value. To examine potential PCR inhibition, a synthetic RNA fragment (RNA Spike I, TATAA Biocenter AB, Goeteborg, Sweden) was spiked into the sample lysates and into only lysis buffer. ΔC_q_ values [calculated by C_q_ RNA Spike (sample lysate) − C_q_ RNA Spike (lysis buffer plus RNA Spike)] of >1 resulted in the exclusion of potentially false‐negative data points. Results of primer pairs showing C_q_ values < 35 in the negative reverse transcription control, results of non‐target amplicons (∆T_m_ [positive control‐sample] > 2°C) and results of dimers [T_m_ < 76.6°C] were excluded from the analysis.

The quantification cycle was defined to be reached at the threshold 0.5 in all cases. CTC expression data were normalized to matched expression data of healthy donor controls (n = 20), described in detail by Keup et al.^33^ Transcripts not exclusively expressed in CTCs but also in the 100‐200 contaminating leucocytes were normalized to the leucocyte‐specific transcript *PTPRC *(∆∆C_q_ = [Cut‐off(gene)‐Sample C_q_(gene)]−[Cut‐off(*PTPRC*)‐Sample C_q_(*PTPRC*). Some transcripts were independent of a growing number of leucocytes (namely *ALK, AR, AURKA, KIT, KRT5, MET, EGFR* and *ERBB3*); thus, the ∆C_q_ value was calculated as follows: ∆C_q_ = [Cut‐off(gene)‐Sample C_q_(gene)]. Only positive Δ(Δ)C_q_ values were regarded as evaluable signals, and signals were only analysed binary. C_q_ values of all patient samples, and healthy donors are listed in Table S1. Data evaluation of the AdnaTest TNBC prototype was described recently in detail elsewhere.^33^

### 2.8 Statistical analysis

Statistical analysis was performed using Winstat (2012.1), an upgrade of Microsoft Excel (www.winstat.de). Survival intervals were screened from the time of CTC analysis until the date of recurrence (PFS) or death (OS) and calculated with Kaplan‑Meier estimator (Log‑rank test). Associations of therapy regimens and gene expression profiles were performed by contingency tables. *P*‑values were generated with the chi‐squared test; if <5 cases were identified in each group, Fisher's exact test was used. *P* < 0.05 was considered to indicate a statistically significant difference. Univariate and multivariate COX‐proportional hazard analysis (Breslow method) was conducted using R version 3.5.1 (2018‐07‐02) including R packages survminer, survival, broom and ggplot2.

## 3 RESULTS

The clinical characteristics of all patients at the time of first diagnosis are shown in Table 1, detailed according to their histological subtypes. The median age of the total group was 51 years (range 27‐86 years) with the majority of patients younger than 60 years (57/75; 76%). About 57% of the patients were postmenopausal, and the predominant histological subtype in all subgroups was invasive ductal carcinoma (72%). Although a difference in grading was obtained in the different subgroups, most TNBC patients (42/51; 82%) and HR−/HER2 + patients (8/10; 80%) had grade III tumours whereas most of the HR+/HER2− patients had a grade II tumour (10/14; 71%), respectively. In all subgroups, most of the patients showed a KI67 above 15% (TNBC 75%; HR+/HER2% − 100%; HR−/HER2 + 90%) and at least 50% of the patients had a KI67 value above 30% (TNBC 67%; HR+/HER2 − 50%; HR−/HER2 + 60%). In general, HR+/HER2− patients mostly presented with T1 tumours (9/14; 64%) and TNBC patients (27/51; 53%) as well as HR−/HER2+ patients (5/10; 50%) with T2 tumours, respectively. At the time of primary diagnosis, 47/75 (63%) of the patients were node‐negative which was mostly reflected in the TNBC (37/51; 73%) and the HR+/HER2− (8/14; 57%) subgroups whereas 80% (8/10) of the HR−/HER+ patients were node‐positive.

Except for one patient in the HR−/HER2+ group, all of the patients received chemotherapy, 92% in the neoadjuvant (69/75) and 7% in the adjuvant (5/75) setting. All patients in the HR+/HER2− subgroup received a neoadjuvant poly‐chemotherapy with taxanes, anthracyclines and cyclophosphamide. 15/51 (20%) of the TNBC patients received carboplatin in addition. All HR−/HER2+ pts received anti‐HER2 treatment (n = 4 Herceptin + Perjeta neoadjuvant, of those n = 3 received the treatment in combination with chemotherapy; n = 1 patient received Herceptin alone; n = 1 received adjuvant chemotherapy with Herceptin; n = 5 received NACT in combination with Herceptin). All patients received Herceptin for the duration of 1 year (not documented in Table 1). Overall, response to therapy resulted in a ratio of 93% (64/69 patients; 46% pCR, 46% pPR) of responders and 7% (5/69) non‐responders with the highest pCR rate found for the HR−/HER2+ subgroup (7/9; 78%) and the lowest in the HR+/HER2− group (4/14; 31%). In patients with TNBC, a pCR was achieved in 45% (21/47) of these patients and 49% of those patients showed a pPR (23/47).

The median follow‐up time was 58.6 months (range: 0‐117.8 months). In the TNBC subgroup, 10/51 (20%) patients had a median PFS time of 30 months (range 2‐57 months) and 8/51 (16%) of the patients died after a median follow‐up time of 19.85 months (range 3‐33 months). In contrast, in the non‐TNBC group, one HR−/HER2+ patient had a relapse after 32 months and one other patient of this subgroup died of a melanoma after 48 months (data not shown).

### 3.1 Gene expression profiles in CTCs of TNBC and non‐TNBC patients before and after therapy

In total, 51 patients with TNBC were analysed for CTCs, 39 BT, 37 AT and 25 pairwise, at both time points. The control group of 24 non‐TNBC patients consisted of 14 HR+/HER2− patients, 12 BT, 13 AT and 11 pairwise as well as 10 HR−/HER2+ patients, 8 BT, 8 AT and 6 pairwise, respectively. The number of analysed genes overexpressed per patient is detailed in Table S2. In patients with TNBC, BT, none of the genes were found overexpressed in only 4/39 (10%) patients, 1‐3 genes were overexpressed in 15/39 (38%) patients and ≥4 genes in 20/39 (51%) patients, respectively. AT, the values were 4/37 (11%), 21/37 (57%) and 12/37 (32%) patients, respectively. In contrast, in the group of non‐TNBC patients, the majority of patients overexpressed only 1‐3 genes (15/20 patients, 75%), BT as well as AT (16/21 patients, 76%).

**Figure 1:**
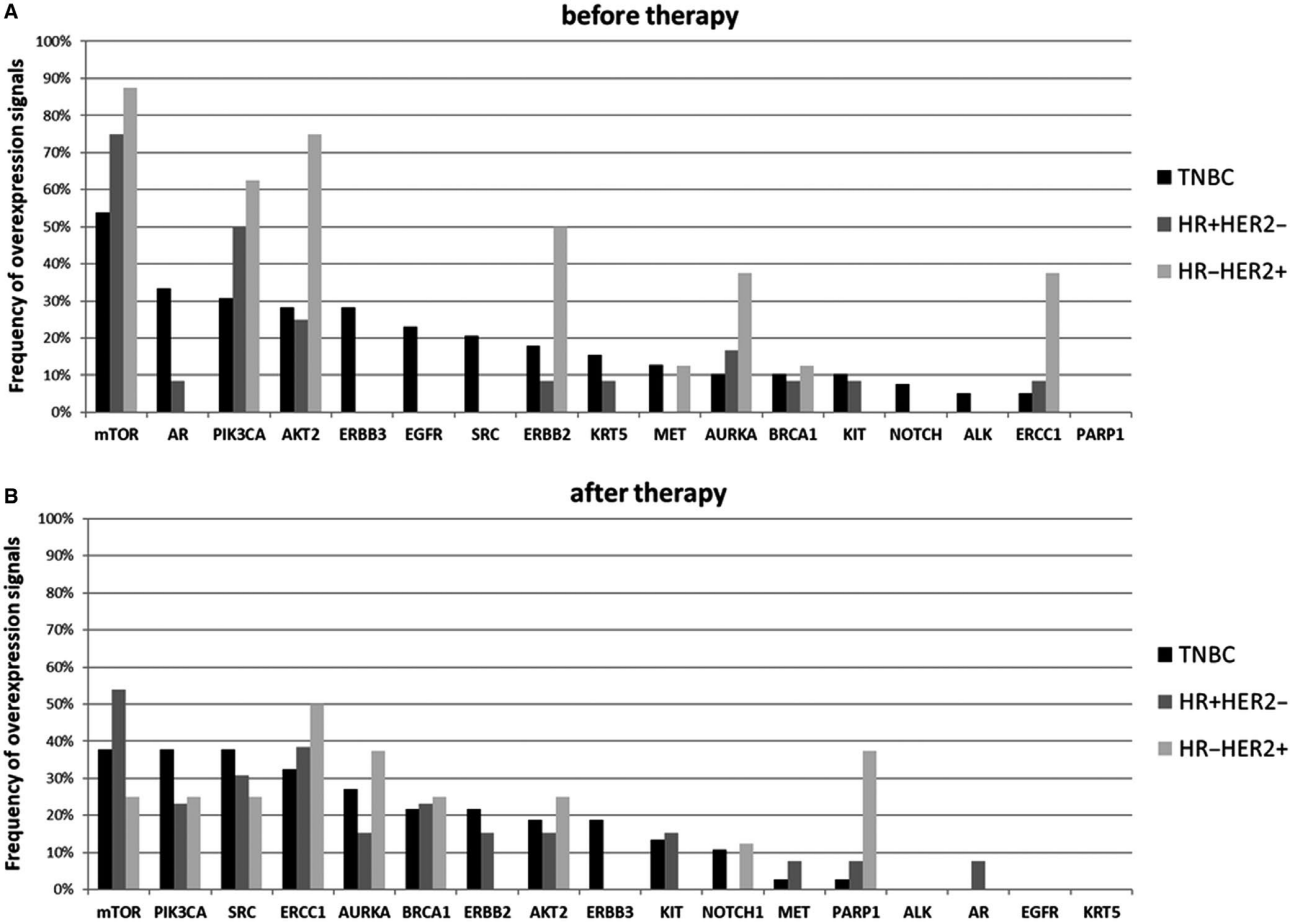
Gene Expression profiles in the different BC subgroups. A, before therapy. B, after therapy. In general, *PIK3CA, AKT2, MTOR* as well as the resistance marker *AURKA* and *ERCC1* were predominantly expressed in all BC subtypes, the latter two genes especially AT. In patients with TNBC, all the different genes were overexpressed BT (except for *PARP1*), probably representing the most heterogeneous CTC population. The most frequently overexpressed genes were *MTOR* (54%), *AR* (33%), *PIK3CA* (31%) and *AKT2* (28%), respectively. A variety of genes including AR (with the exception of one positive patient in the HR+/HER2− group), *ERBB3* (28%), *EGFR* (23%), *SRC* (21%), *NOTCH* (8%) and *ALK* (5%) were uniquely overexpressed in patients with TNBC BT

Gene expression profiles for every BC subgroup BT and AT are documented in detail in Figure 1. In general, *PIK3CA, AKT2, MTOR* as well as the resistance markers *AURKA* and *ERCC1* were predominantly expressed in all BC subtypes, the latter two genes especially AT. The frequency of overexpression signals was lower in all subgroups AT. In patients with TNBC, all the different genes were overexpressed BT (except for *PARP1*), probably representing the most heterogeneous CTC population. The most frequently overexpressed genes were *MTOR* (54%), *AR* (33%), *PIK3CA* (31%) and *AKT2* (28%), respectively. A variety of genes including AR (with the exception of one positive patient in the HR+/HER2− group), *ERBB3* (28%), *EGFR* (23%), *SRC* (21%), *NOTCH* (8%) and *ALK* (5%) were uniquely overexpressed in patients with TNBC BT. AT, *MTOR* (38%), *PIK3CA* (38%), *SRC* (38%), *ERCC1* (32%) and *AURKA* (27%) were still predominantly overexpressed whereas the overexpression of *ALK, AR, EGFR* and *KRT5* could not be detected any longer in patients with TNBC. Particularly, *ERBB2+ *and *ERBB3 + *CTCs were found at both time points in about 20% of the patients.

In non‐TNBC patients, BT, the overexpression pattern in CTCs of HR+/HER2− patients was, although to a lower extend, similar to the profile detected in HR−/HER2+ patients. The overexpression of *SRC* seemed to be induced by therapy in both non‐TNBC subgroups (31% in the HR+/HER2− and 25% in the HR‐HER2+ group) whereas *NOTCH* (13%) and *PARP1* (38%) were mainly detected in the group of HR−/HER2+ patients AT. Especially, in this group of patients, *ERBB2 + *CTCs, initially detected in 50% of the cases, completely disappeared AT, most likely due to anti‐HER2 targeted treatment which seemed to also markedly reduce initial *PI3K/AKT/MTOR* overexpression. Notably, AT, *AURKA + *CTCs and *ERCC1* + CTCs were found in 38% and 50% of HR−/HER2 + cases and in 15% and 38% of the HR+/HER2− patients.

### 3.2 Gene expression profiles in CTCs of TNBC and non‐TNBC patients before and after therapy (pairwise)

**Figure 2:**
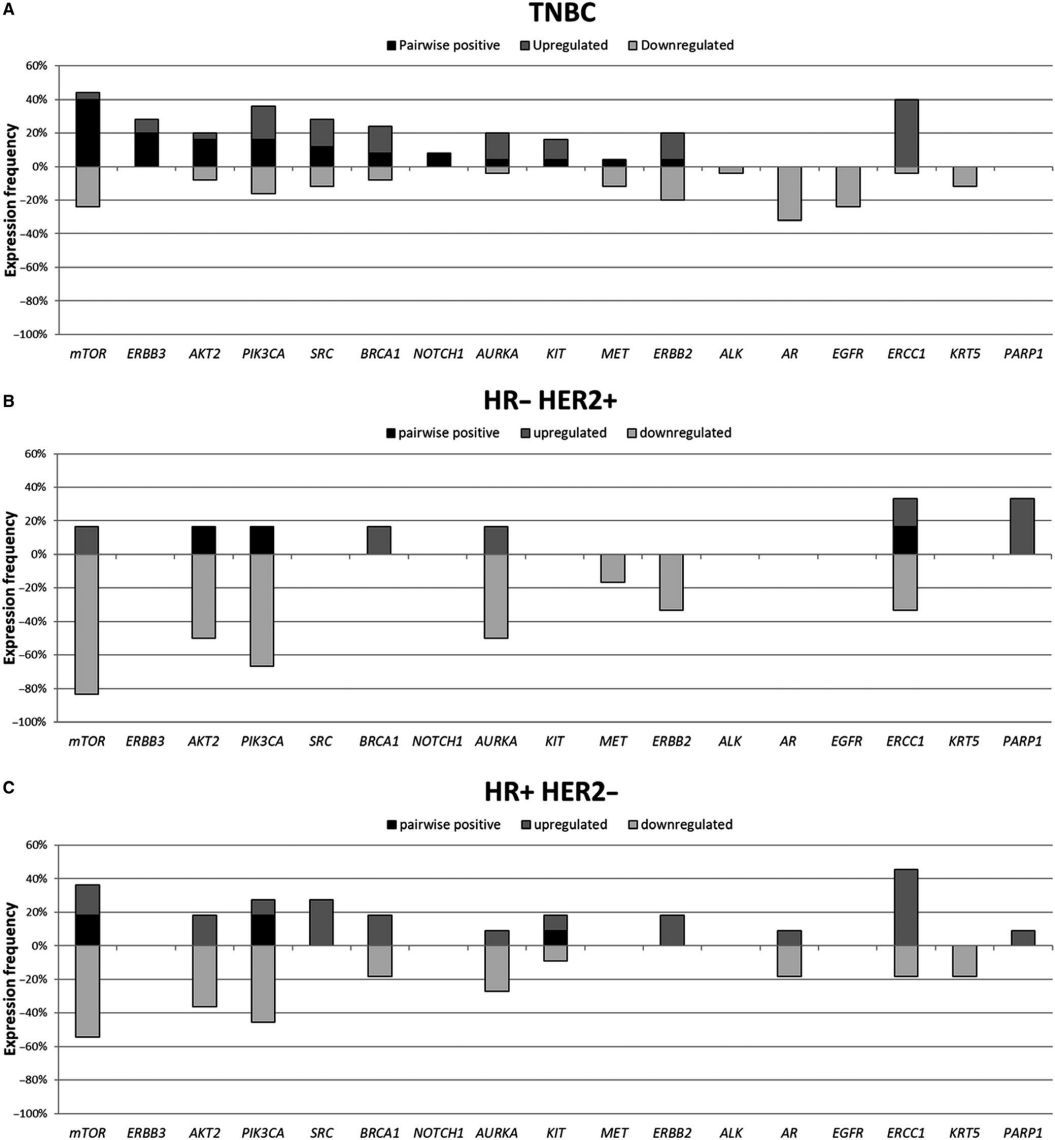
Pairwise gene expression in all BC subgroups before and after therapy. A, TNBC. B, HR+/HER2− BC. C, HR−/HER2 + BC. In patients with TNBC (A), 11 (65%) of the 17 analysed genes were overexpressed at both time points, predominantly *MTOR*, *ERBB3, AKT2* and *PIK3CA*. In the group of non‐TNBC patients, only 3/17 (18%) genes were detected BT as well as AT (*MTOR, PIK3CA* and *KIT* in the HR+/HER2− group (B) and *PIK3CA, AKT2* and *ERCC1* in the HR−/HER2+ (C) group, respectively). Although the number of analysed pairs in these two subgroups of non‐TNBC patients was quite small, in HR+/HER− patients, a comparable number of genes were differentially up‐ (n = 11) or down‐regulated (n = 9) whereas in the HR−/ HER2 + group, most of the genes expressed BT were down‐regulated AT (n = 7)

Pairwise expression of the different genes BT and AT is detailed in Figure 2. In patients with TNBC, 11 (65%) of the 17 analysed genes were overexpressed at both time points, predominantly *MTOR*, *ERBB3, AKT2* and *PIK3CA*. In the group of non‐TNBC patients, only 3/17 (18%) genes were detected BT as well as AT (*MTOR, PIK3CA* and *KIT* in the HR+/HER2− group and *PIK3CA, AKT2* and *ERCC1* in the HR−/HER2+ group, respectively). Although the number of analysed pairs in these two subgroups of non‐TNBC patients was quite small, in HR+/HER− patients, a comparable number of genes were differentially up‐ (n = 11) or down‐regulated (n = 9), whereas in the HR−/HER2+ group, most of the genes expressed BT were down‐regulated AT (n = 7).

### 3.3 Survival analysis

Survival analysis with regard to PFS and OS was only feasible for the group of TNBC patients with 10 relapses after a median follow‐up time of 30 months (range 2‐57 months) and eight deaths, six of them BC‐specific, after a median follow‐up time of 19.85 months (range 3‐33 months), respectively. In the group of non‐TNBC patients, only two events were documented. One HR−/HER2+ patient died of a melanoma and one patient of this subgroup, treated with Herceptin and Perjeta, relapsed after 32 months. Particularly in this patient, *ERBB2 + *and *ERBB3 + *CTCs were not detected at any time point and *AURKA* and *ERCC1* were the only marker expressed BT and *ERCC1* and *MTOR* AT, respectively.

**Figure 3:**
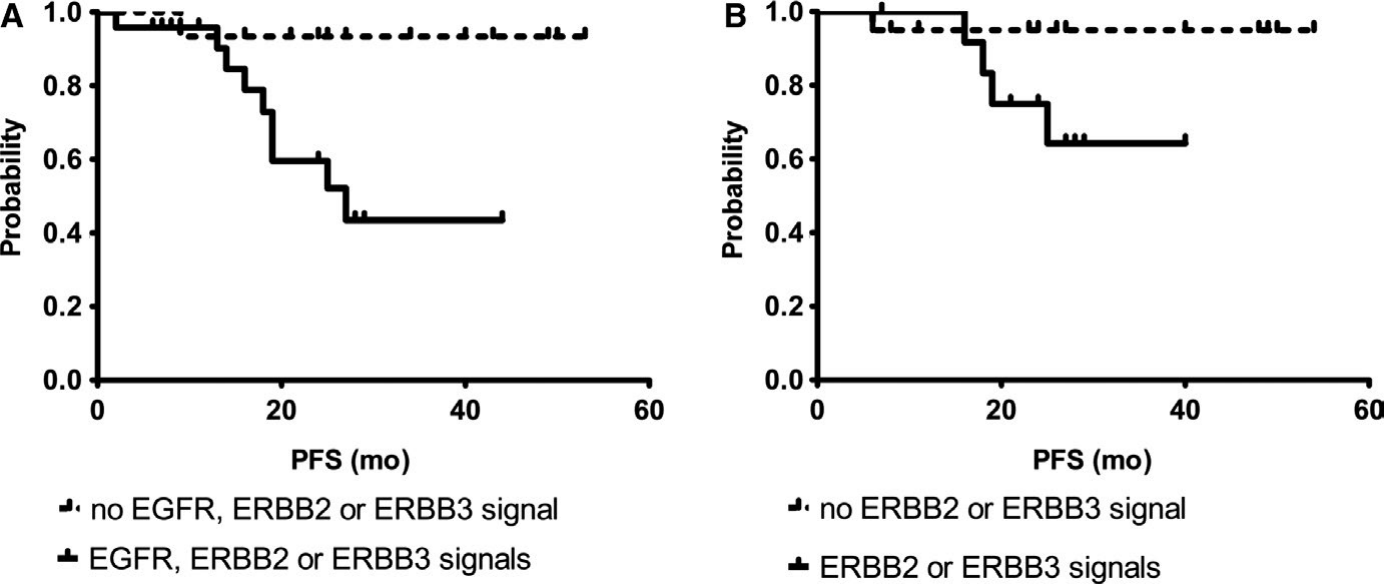
Survival Correlations for CTCs Expressing *EGFR/ERBB2/ERBB3*. A, Progression‐free survival before therapy. B, Progression‐free survival after therapy. Survival analysis with regard to PFS was only feasible for the group of TNBC patients with 10 relapses after a median follow‐up time of 30 mo (range 2‐57 mo). The presence of *EGFR+ *or *ERBB2+ *or *ERBB3 + *CTCs (A) in patients with TNBC BT and (B) *ERBB2 + *or *ERBB3 + *CTCs AT significantly correlated with a reduced PFS (*P* = 0.01; *P* = 0.02)

Of all 18 panel genes investigated, only ERCC1 and the combined ‘all ERBB family status’ including EGFR, ERBB2 and ERBB3, profiles were correlated with PFS before therapy in COX univariate proportional hazard analysis (detailed in Figure S1). As shown in Figure 3A and B, the presence of *EGFR+ *or *ERBB2+ *or *ERBB3 + *CTCs in TNBC patients BT and *ERBB2+/ERBB3 + *CTCs AT significantly correlated with a reduced PFS (0.01 and *P* = 0.02). Interestingly, the presence of *ERBB3 + *CTCs alone BT was sufficient to significantly (*P* = 0.04) indicate a shorter PFS (data not shown). OS analysis did not reach statistical significance.

**Figure 4:**
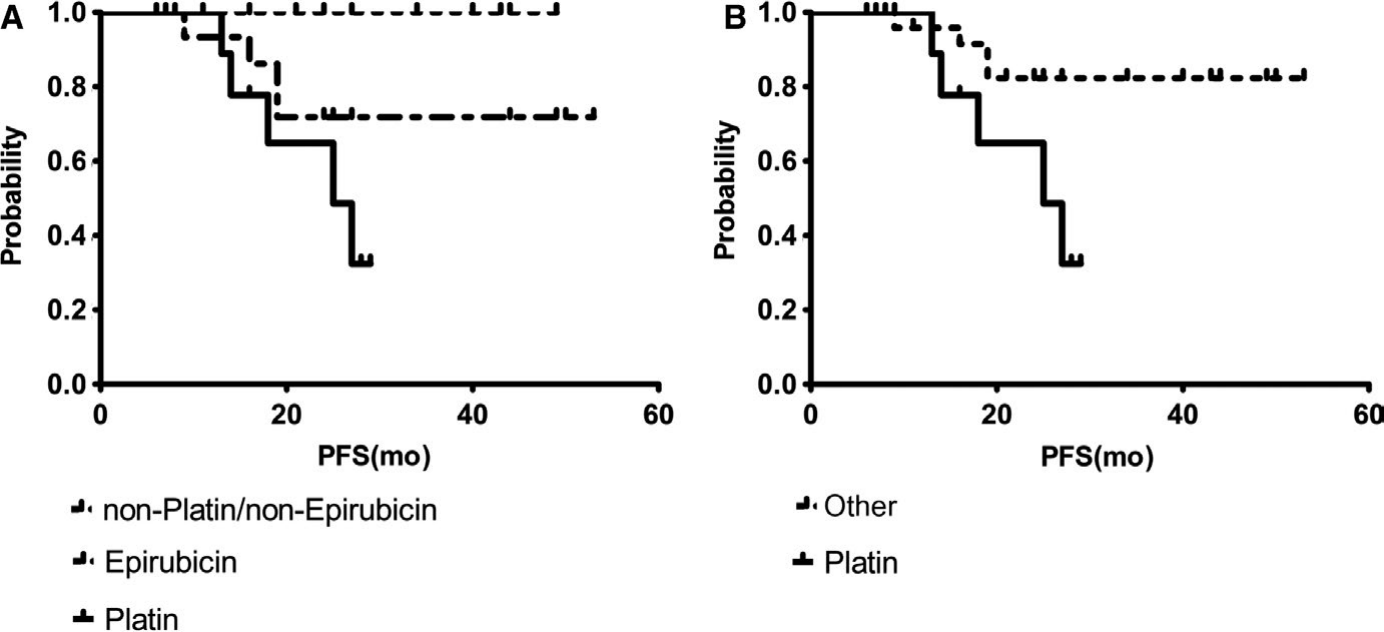
Survival Correlations with regard to different chemotherapy drugs—progression‐free survival. A, Platinum‐ or epirubicin‐containing therapy or others. B, Platinum‐ vs non‐platinum containing therapy. The relationship between PFS and the given therapy is detailed. Patients receiving platinum‐based therapy or epirubicin had a significantly shorter PFS (*P* = 0.03) than patients who received other therapeutic regimens (A). Survival correlations for platinum vs non‐platinum containing therapies resulted in a *P*‐value of 0.02 (B)

The relationship between PFS and the given therapy is shown in Figure 4A and B. Patients receiving platinum‐based therapy or epirubicin had a significantly shorter PFS (*P* = 0.03) than patients who received other therapeutic regimens (Figure 4A). Survival correlations for platinum vs non‐platinum containing therapies resulted in a *P*‐value of 0.02 (Figure 4B). When gene expression profiles were correlated with platinum‐based chemotherapy, significant correlations were only found for *PIK3CA* overexpression in CTCs AT (*P* = 0.01) but not BT (0.51), probably resulting in the expression of genes related to resistance (*BRCA1* [*P* = 0.03], *ERCC1* [*P* = 0.03], *NOTCH1* [0.02]) (data not shown).

**Figure 5:**
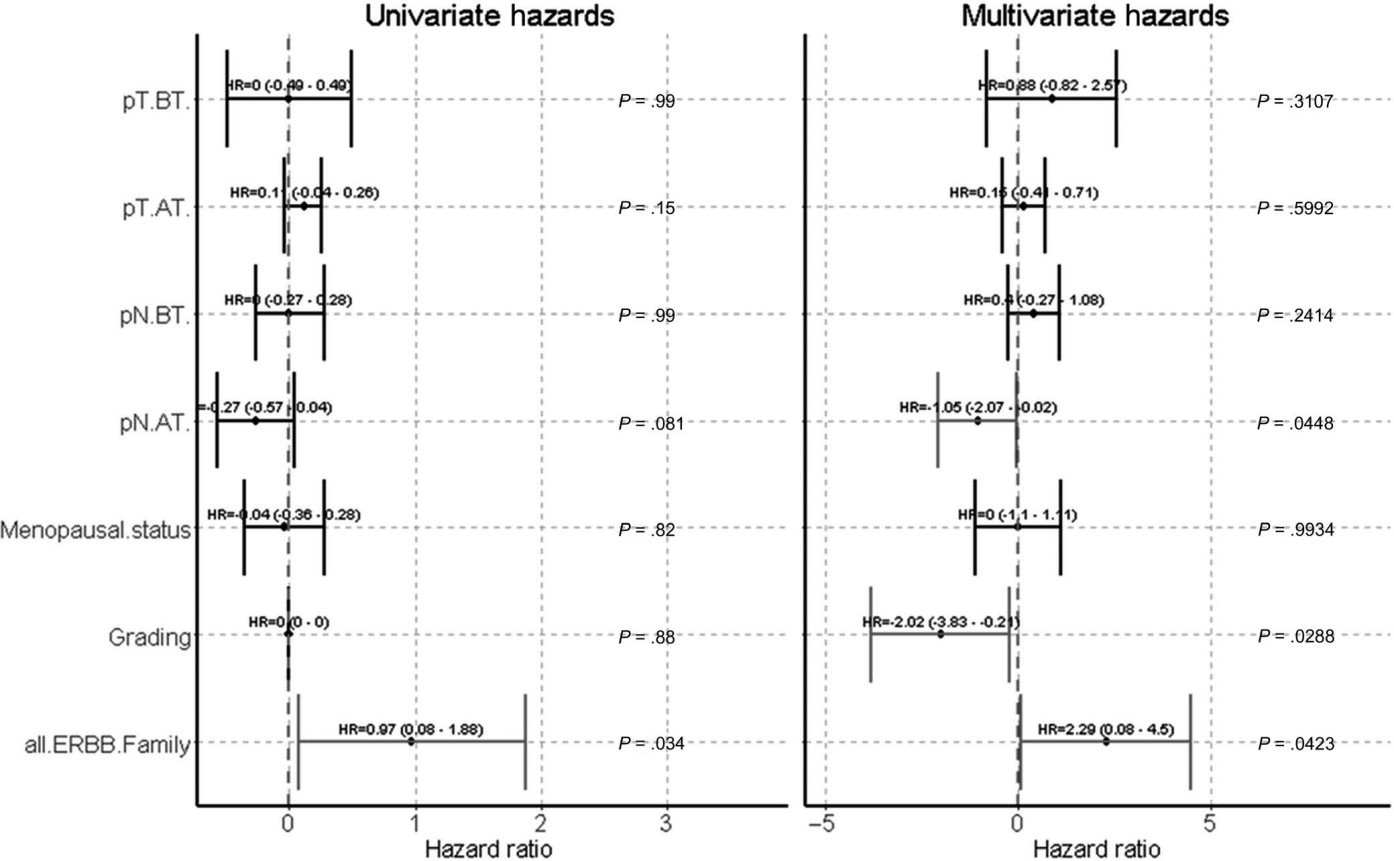
Univariate and multivariate Cox‐Hazards for PFS. Using COX multivariate analysis with standard staging parameters like nodal stage and tumour size before and after therapy as well as menopausal status and grading, the CTC ‘all ERBB family status’ turned out as a significant, independent unfavourable predictor for PFS (*P* = 0.042). c (clinical)/p (pathological) T (tumour), c/pN (node), BT (before therapy), AT (after therapy)

Using COX multivariate proportional hazard analysis with standard staging parameters like nodal stage and tumour size BT and AT as well as menopausal status and grading, the CTC ‘all ERBB family status’ turned out as a significant, independent unfavourable predictor for PFS (Figure 5).

## 4 DISCUSSION

We here demonstrated that markers representing the *PIK3CA* signalling pathway as well as the resistance marker *ERCC1* and *AURKA* were predominantly present in all BC subtypes, the two latter ones especially AT. In CTCs derived from patients with TNBC, the expression of all genes contained in the 17 gene panel was observed, thereby representing the most heterogeneous CTC population for this stem cell/EMT‐enriched panel of genes. Furthermore, TNBC‐derived CTCs appeared to up‐regulate most of the 17 genes or kept their expression frequency on a high level after therapy except for *AR* and *EGFR*. This involved all genes related to the PIK3CA pathway, all resistance related genes (*BRCA1, AURKA, ERCC1*) and *ERBB3*. Interestingly, *ERBB2* was differentially expressed BT as compared to expression levels AT but stayed on a level of 20% which might be regarded high considering an initially HER2‐negative tissue. *EGFR+*/*ERBB2+/ERBB3 + *CTCs BT and *ERBB2+/ERBB3 + *CTCs AT further indicated worse prognosis, and interestingly, platinum‐based therapy was related to a reduced PFS and further correlated with CTC *PIK3CA* overexpression AT but not BT which probably resulted in the expression of genes related to resistance. Furthermore, the CTC ‘all ERBB family status’ turned out as a significant, independent unfavourable predictor for PFS.

In non‐TNBC patients, we demonstrated that in CTCs from HR−/HER2+ patients most of the 17 genes were down‐regulated AT, most likely because of anti‐HER2 therapy which was the common treatment choice in that group. *ERBB2 + *CTCs were no longer detected after treatment but also genes involved in the PIK3CA signalling pathway as well as the resistance markers were strongly affected. In the HR+/HER2− group, the PIK3CA signalling pathway also got down‐regulated but some of the genes appeared de novo (*SRC, ERBB2*) and *ERCC1* was strongly up‐regulated which might be considered as an onset of resistance.

Although the heterogeneity of CTCs has widely been confirmed, only a very few studies has characterized CTCs in primary and metastatic TNBC patients, up to now. Using confocal laser scanning and microscopy for immunofluorescent staining of ER, PR, HER2 and EGFR, triple‐staining experiments revealed that distinct CTC subpopulations could be identified in individual patients. CTCs in early‐stage TNBC were shown to frequently express ER, PR and HER2, but predominantly EGFR.^31^ These data, including the frequency of marker expression, are concordant with our findings for *ERBB2* and especially *EGFR*. Besides the presence and persistence of a variety of genes in that BC subtype, probably leading to worse outcome, we were further able to show that *EGFR+/ERBB2+/ERBB3 + *CTCs BT as well as *ERBB2+/ERBB3 + *CTCs AT were a strong predictor for a reduced PFS, with a dominating influence of *EGFR* and *ERBB3* BT but *ERBB2* and *ERBB3* AT. Notably, even the presence of *ERBB3 + *CTCs alone BT was sufficient to indicate a shorter PFS. In contrast to the data shown here, the paper by Kruijff et al, describing AR expression in CTCs of metastatic breast cancer patients using the CellSearch system, thus, selecting EpCAM‐positive CTCs for further *AR* mRNA expression analysis, detected *AR + *CTCs in only 13% of TNBC cases. We can confirm this low rate in a group of metastatic TNBC patients of our Department (other patients than presented here) that we had recently analysed (unpublished data). Furthermore, in patients with HR+/HER2− tumours (n = 75), the detection rate was 36% in the paper by Kruijff et al, which is also comparable with our recently published data,^33^ detecting *AR + *CTCs in more than 20% of the 35 analysed HR+/HER2− patients in the follow‐up of the disease. In our primary TNBC cohort, *AR + *CTCs were only present BT, but rarely AT. Thus, tumour therapy might have eliminated these cells.^34^

A variety of studies has already proven a discrepancy between ER, PR and HER2 on the primary tumour and metastatic tissue ^35^ as well as on single tumour cells and tumour tissue, especially for HER2.^25^, ^26^, ^27^, ^36^, ^37^ These cases might eventually benefit from anti‐HER2‐targeted therapy as observed in the corresponding cases in the HR−/HER2 + cohort where *ERBB2 + *CTCs initially detected in 50% of the patients, completely disappeared after anti‐HER2‐targeted treatment. A few pilot studies have already demonstrated that anti‐HER2‐targeted therapy was able to successfully eliminate HER2‐positive single circulating and disseminated tumour cells in non‐metastatic and metastatic BC.^38^, ^39^, ^40^ Furthermore, the treatment regimens in this subgroup of patients also seemed to markedly reduce initial *PIK3CA/AKT2/MTOR* gene expression, a pathway frequently involved in maintaining stem cell and EMT character of tumour cells^41^ which might lead to a better outcome in this subgroup of non‐TNBC patients. Moreover, *NOTCH, PARP1* and *SRC*, associated with differentiation, proliferation and tumour transformation in BC,^42^ seemed to be induced by therapy in about 12%, 38% and 22% of these patients, respectively. Further follow‐up is needed to clarify whether these markers might be predictive for relapse and whether PARP inhibitors could probably be successful in eliminating these CTCs.^43^

For TNBC, the role of EGFR but also HER3 has often been discussed.^44^ Although the expression of *EGFR* could frequently be observed in TNBC tumour tissue, the success of EGFR‐targeted regimens was reported, so far, to be insufficient in terms of sensitivity. It is discussed that molecular mechanisms other than EGFR alone must be considered to develop successful combination therapies. Our finding that all *EGFR* + CTCs BT disappeared after treatment might point to the direction that initial pathways can be switched to alternatives of which *ERBB3* or de novo expressed *ERBB2* AT could be an indicator for. In that context, cell culture experiments demonstrated that the efficiency of EGFR and PIK3CA inhibitors in TNBC cells was decreased by activating *ERBB3* via heregulin.^45^ They further showed an elevated presence of HER3 in residual tumour tissue of TNBC patients not having achieved a pCR after therapy with EGFR‐targeted antibodies.

Independent of the detected CTC profiles, we surprisingly found that patients receiving platinum‐ or epirubicin‐containing regimens had a significantly worse PFS compared with those patients receiving other therapies. Although TNBC has been shown to be sensitive to DNA‐damaging platinum‐based agents,^12^ however, the results in several clinical studies were contradictory. However, the addition of carboplatin in the phase II GeparSixto Trial resulted in a significant improved 3‐year PFS,^8^ EFS and OS in another randomized phase II trial conducted by the Cancer Leukemia Group (CALGB 40603) with a median follow‐up duration of 39 months, demonstrated that treatment with carboplatin increased the pCR rate but did not significantly affect PFS and OS.^7^ Patients who achieved a pCR in our study also had an improved PFS and OS which is in accordance with the results of other already published clinical studies.^2^, ^3^ Thus, to decide whether platinum‐based chemotherapy is the treatment of choice in this subgroup and based on our findings that CTC RNA profile changes correlated with the response to platinum treatment, the characterization of CTCs, therefore, could provide valuable information about platinum resistance in TNBC.

Based on our current results, showing that platinum‐based chemotherapy significantly correlated with the PIK3CA expression in CTCs AT but not before treatment, one may speculate that platinum‐based therapy induces EMT in CTCs via activation of *PIC3CA*, further leading to the induction of other resistance mechanisms. For cisplatin‐based therapies, the induction of EMT has been comprehensively described^46^, ^47^ as well as further correlations with regard to resistance genes like *ERCC1*, regulated by Snail in head and neck squamous cell carcinoma.^48^ The role of *BRCA1*, correlated with *PIK3CA* expression in this study, has generally been described to be involved in DNA repair mechanisms and cell cycle regulation which explain its role in counteracting DNA‐damaging chemotherapy.^49^

In general, in all BC subtypes, genes associated with resistance, especially *AURKA* and *ERCC1,* were frequently expressed, mostly AT, perhaps representing one of the major problems in cancer treatment. We recently demonstrated that CTCs detected after NACT were associated with tumour stem cell characteristics as well as *ERCC1* expression which may also suggest a potential selection of this CTC subset by chemotherapy.^23^

## 5 CONCLUSIONS AND LIMITATIONS OF THE STUDY

Taking all these considerations into account, a comprehensive characterization of CTCs, probably on the single cell level, might help to identify patients for further targeted therapy. However, we would like to emphasize that this is a small ‘proof of principle study’ which has to be confirmed in a larger study group. In this context, also statistical analysis has an exploratory character; thus, multiple testing correction (eg a Bonferroni correction) was not applied but will be considered in further confirmatory settings.^50^

## CONFLICT OF INTEREST

The authors confirm that there are no conflicts of interest.

## AUTHOR CONTRIBUTIONS

AKB contributed to protocol/ project development, patient recruitment, data collection, data analysis and manuscript writing; CK contributed to method evaluation and visualization of the results; OH contributed to protocol/ project development, patient recruitment, data collection, data analysis and manuscript writing; SH contributed to data analysis and establishment of multimarker panel; RK contributed to overall responsibility; SKB contributed to protocol/ project development, data collection, data analysis, manuscript writing.

## ETHICAL APPROVAL

All specimens were obtained and collected after written informed consent from all subjects using protocols approved by the clinical ethic committee of the University Hospital Essen (05/2856).

## CONSENT FOR PUBLICATION

Not applicable.

## Supporting information

Fig S1

Tab S1

Tab S2

## Data Availability

C_q_ values of the patient samples (in duplicate) and healthy donors are listed in Table S1. Further information is available from the corresponding author on reasonable request.
