# Supplementary figures and images for: Molecular characterization of circulating tumour cells identifies predictive markers for outcome in primary, triple‐negative breast cancer patients
Source: J Cell Mol Med. 2020 Jun 18;24(15):8405–16. doi: 10.1111/jcmm.15349 (PMC7412423; doi:10.1111/jcmm.15349)

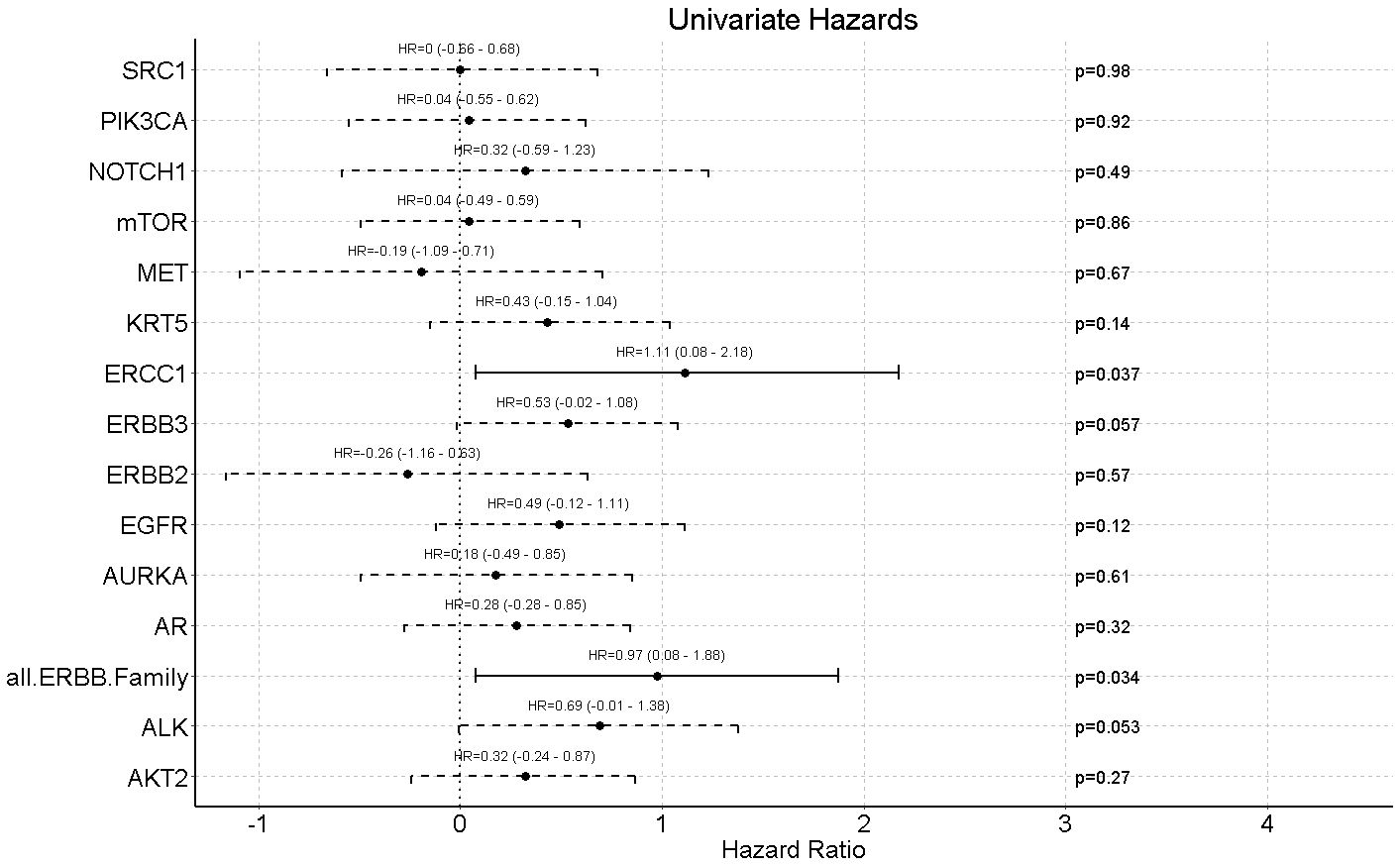

Supplement: Supplementary file 1 — Fig S1 [file JCMM-24-8405-s001.tiff]
