# Supplementary material for: Molecular characterization of circulating tumour cells identifies predictive markers for outcome in primary, triple‐negative breast cancer patients
Source: J Cell Mol Med. 2020 Jun 18;24(15):8405–16. doi: 10.1111/jcmm.15349 (PMC7412423; doi:10.1111/jcmm.15349)
Supplement: Supplementary file 3 — Tab S2 [file JCMM-24-8405-s003.docx]

**Suppl Table 2.** Number of genes overexpressed per patient.

| **Before Therapy After Therapy** | | | | |
| --- | --- | --- | --- | --- |
| **No of genes**  **expressed**  **per patient** | **TNBC**  **pts** | **Non-TNBC**  **pts** | **TNBC**  **pts** | **Non-TNBC**  **pts** |
| **0** | **4/39 (10%)** | **0/20 (0%)** | **4/37 (11%)** | **1/21 (5%)** |
| **1-3** | **15/39 (38%)** | **15/20 (75%)** | **21/37 (57%)** | **16/21 (76%)** |
| **> 4** | **20/39 (51%)** | **5/20 (25%)** | **12/37 (32%)** | **4/21 (19%)** |
